# Temperature dependence of the single photon emission from interface-fluctuation GaN quantum dots

**DOI:** 10.1038/s41598-017-16040-x

**Published:** 2017-11-23

**Authors:** F. Le Roux, K. Gao, M. Holmes, S. Kako, M. Arita, Y. Arakawa

**Affiliations:** 1 Institute of Industrial Science, The University of Tokyo, 4-6-1 Komaba, Meguro-ku, Tokyo, 153-8505 Japan; 2 Institute for Nano Quantum Information Electronics, The University of Tokyo, 4-6-1 Komaba, Meguro-ku, Tokyo, 153-8505 Japan

## Abstract

The temperature dependent single photon emission statistics of interface-fluctuation GaN quantum dots are reported. Quantum light emission is confirmed at temperatures up to ~77 K, by which point the background emission degrades the emission purity and results in a measured g^(2)^ (0) in excess of 0.5. A discussion on the extent of the background contamination is also given through comparison to extensive data taken under various ambient and experimental conditions, revealing that the quantum dots themselves are emitting single photons with high purity.

## Introduction

Sources of single photons will be of use for several future quantum technologies, and are therefore an active topic of intense academic research and development^1^. Although there are several technologies that can be used for the emission of single photons^2–7^, in recent years III-nitride based quantum dots (QDs) have been the subject of increased attention due to their possible operation over a wide range of wavelengths from the ultraviolet to the red end of the visible spectrum^8–15^. Furthermore, III-nitride QD-based single photon sources have also been shown to operate at room temperature^13,14^ and even at elevated temperatures^16^. However, being a comparatively novel material system, typically-grown material exhibits high densities of crystal defects and charge traps (~10^17^–10^19^cm^−3^), the rapid charging and de-charging of which leads to an undesirable spectral diffusion effect and a related broadening of the emission linewidth^17–19^. This effect is exacerbated in wurtzite III-nitride quantum dots due to an internal electric field which induces a static dipole moment in confined excitons and results in a strong interaction with the environment^20,21^, even for dots with a relatively small extension in the direction of the field^19^. Following in the footsteps of III-As QD research^22^, recently, interface fluctuation GaN quantum dots have been developed^23^ that exhibit narrow linewidths compared to typical GaN QDs^12,18^. These interface-fluctuation quantum dots, so-called due to their formation as charge localization centers at positions of thickness fluctuation in quantum wells^22,23^, emit in the near ultraviolet at wavelengths of ∼350 nm (see ref.^23^ for detailed growth details). In this paper we report the temperature dependence of the single photon emission properties of these dots with regards to spectral contamination from an unavoidable background emission.

**Figure 1:**
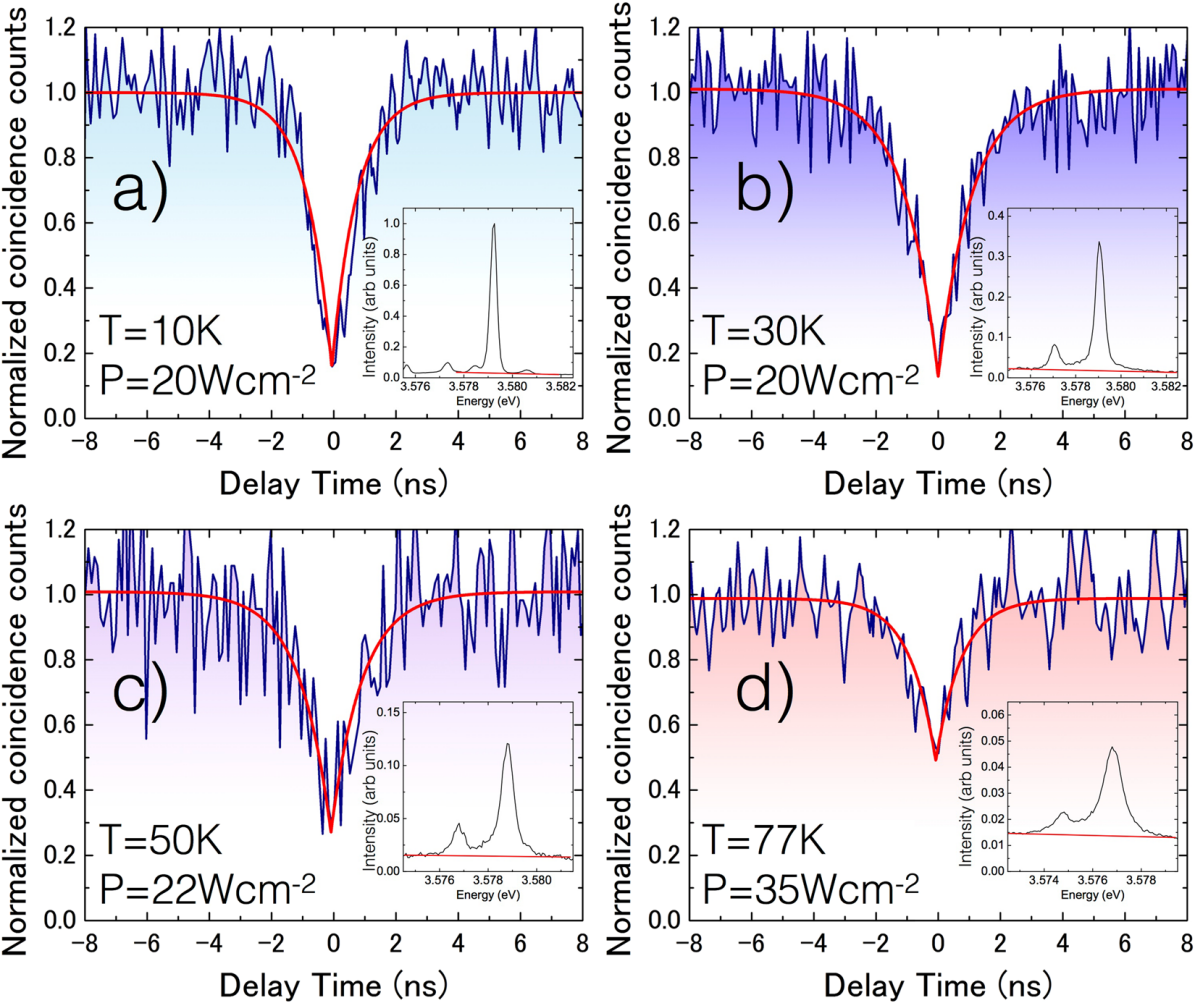
(**a**–**d**) *g* ^(2)^ (τ) measurements (and corresponding emission spectra) of the emission from a single GaN interface-fluctuation quantum dot at temperatures between 10 K and 77 K.

The principle of the experimental study reported here was to ascertain the temperature limit at which single photon emission could be observed from this particular type of novel III-nitride quantum dot, as grown. To that end, the sample was held in a continuous-flow liquid helium cryostat equipped with a temperature controller, and measurements of the second order intensity autocorrelation at time delay τ:

1 $${g}^{(2)}(\tau )=\frac{\langle I(t)\cdot I(t+\tau )\rangle }{\langle I{(t)}^{2}\rangle }$$

were performed at increasingly higher temperatures until the value of *g* ^(2)^ (0) became larger than 0.5 (the upper limit at which a measurement of single photon emission can be claimed). Measurements were performed under CW excitation using a diode-pumped solid state laser operating at a wavelength of 266 nm. Excitation was carried out at a steep axis, and the resulting photoluminescence was collected using a 40x objective lens operational in the near UV (NA 0.6). The signal was then filtered spatially with a confocal pinhole to help isolate the emission from a single structure, and the emission from the selected area was guided to a 70 cm single-grating spectrometer equipped with a 2400 lmm^−1^ grating and a liquid nitrogen cooled CCD detector. The photon emission from an isolated quantum dot could then be directed through the exit slit of the spectrometer (matched to the emission linewidth), whereby the intensity autocorrelation could be probed using a typical Hanbury Brown & Twiss type setup consisting of a beam splitter, two photomultiplier tubes (PMTs), and timing electronics. Temperature dependent *g* ^(2)^ (τ) measurements, along with corresponding emission spectra, are shown and summarized in Fig. 1a–d.

All measurements were performed just below the saturation point of the emission intensity (corresponding to excitation powers of order 0.02 kWcm^−2^, see the figure for exact values). The measured values of *g* ^(2)^ (0) were extracted by fitting the normalized experimental data with a typical exponential function as shown by the red curves in the figure.

Whilst the measured *g* ^(2)^ (0) values are <0.2 for T < 30 K - clear evidence of single photon emission- it is apparent that as the temperature is raised, the emission intensity of the dot reduces significantly (by approximately one order of magnitude when the temperature is raised to 77 K), and a broad background emission (indicated by red lines in the displayed emission spectra) increasingly imposes itself relatively upon the emission spectrum. The presence of this background emission degrades the purity with which the single photon emission can be collected from the QD. Indeed, we measure a *g* ^(2)^ (0) value of 0.52 at a temperature of ∼77 K, which, although being evidence of the sub-Poissonian statistics of the collected light (and hence it’s quantum nature), is not sufficient to claim the isolated emission of single photons from the system.

**Figure 2:**
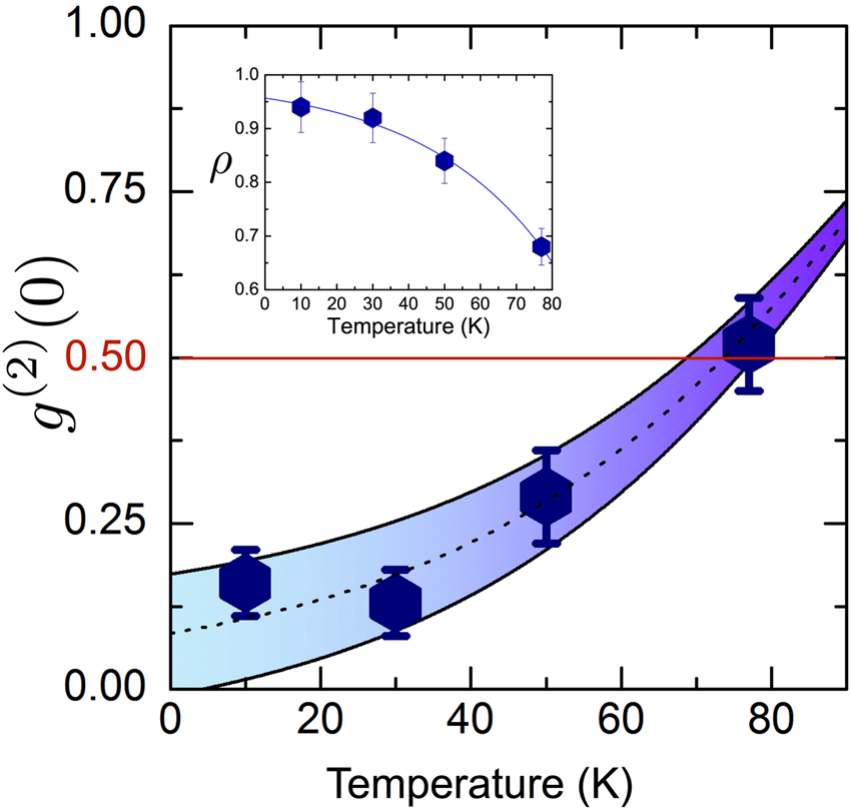
Temperature dependence of the measured *g* ^(2)^ (0) values (data points), along with an extrapolated curve of the background spectral contamination limited values, *g*_*l*_^(2)^ (0) (dashed line). The upper and lower bounds account for a ±5% error in *ρ*. The figure inset shows the extracted values of *ρ* and the exponential fitting to show its temperature dependence.

Whilst it is the measurement of *g* ^(2)^ (0) that gives us the meaningful information of how well the system (including the quantum dot, surrounding material, and excitation/collection optics) is capable of providing single photons (or how purely the emission from a single quantum emitter can be extracted), it is instructive to investigate the extent to which the background emission is actually responsible for the degradation of the measured *g* ^(2)^ (0) values. When taking into account the effect of uncorrelated background-emission on an otherwise pure single photon emitter, it can be shown that the measured *g* ^(2)^ (0) will be limited to a value of

2 $${g}_{l}^{(2)}(0)=1-{\rho }^{2},$$

where *ρ* is the ratio between the measured single photon emission intensity and the total measured signal (including the background emission)^24^. This simple expression puts a lower limit on the value of *ρ* required for the experimental verification of single photon emission, namely $\rho  > 1/\sqrt{2}$. In order to evaluate the effect of the background emission, we calculated the values of *ρ* from the measured spectrum and extrapolated its temperature dependence phenomenologically via fitting with an exponential function as shown in the Fig. 2 inset. In Fig. 2 we plot the measured *g* ^(2)^ (0) values as a function of temperature compared to the background limited values, where the dashed line in the figure is the background limited *g*_*l*_^(2)^ (0) value extrapolated from the data, and the upper and lower bounds allow for a ±5% error in *ρ*. It is clear, from the fact that the measured data points lie along the temperature dependent curve of *g*_*l*_^(2)^ (0), that the background emission is indeed the limiting factor of the single photon emission in this case, leading to a temperature limit of these QDs, in their current form, of ∼77 K. It is important to note that while this result may seem trivial at first glance, it is in contrast to many other examples of III-nitride QDs in the literature, in which such ‘background correction’ does not necessarily result in corrected *g* ^(2)^ (0) values of zero (possibly due to the experimentally temporally-unresolved measurement of a rapid successive emission of two photons, or indeed intermittent multiple photon emission)^14,25–30^. In the present case, the fact that the background is the sole cause of the *g* ^(2)^ (0) degradation shows that the quantum dots themselves are acting as pure single photon emitters.

**Figure 3:**
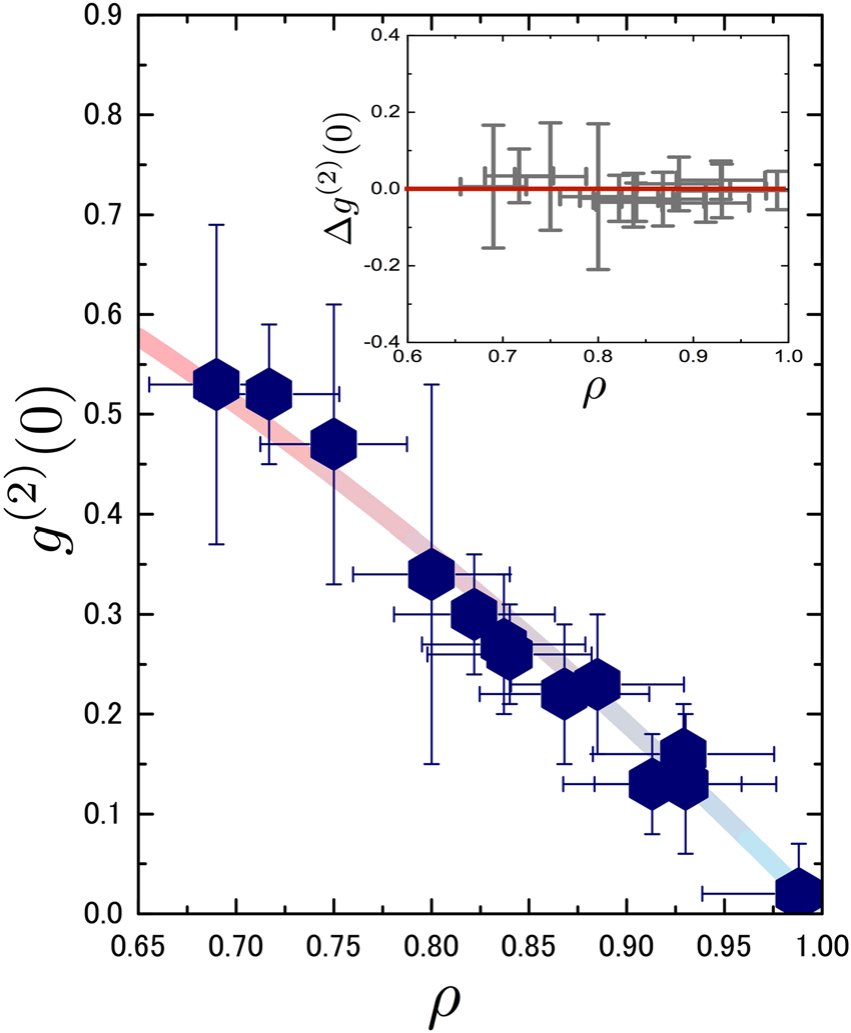
Measured *g* ^(2)^ (0) values from several quantum dots under various ambient conditions shown as a function of the measured value of *ρ*. The solid line is a plot of *g*_*l*_^(2)^ (0) showing clearly that to within the experimental error the background emission entirely accounts for the non-zero measurements of *g* ^(2)^ (0). The figure inset shows the residual values of ${\rm{\Delta }}{g}^{(2)}(0)$.

Finally, in order to further confirm the extent of the background emission limitation, several measurements were made of a number of different quantum dots under various excitation and ambient conditions. The measured *g* ^(2)^ (0) values are shown in Fig. 3 as a plot against the values of *ρ* extracted from the spectra. It is clear, to within the experimental error for all measurements, that the measured data points follow the expected curve for a pure single photon emitter combined with uncorrelated background emission i.e:$\,{g}^{(2)}(0)={g}_{l}^{(2)}(0)$. That is, to within the experimental error, the background emission entirely accounts for the measured non-zero values of *g* ^(2)^ (0). This is further exemplified by the figure inset, in which the residuals between the measured and background limited values, ${\rm{\Delta }}{g}^{(2)}(0)={g}^{(2)}(0)-{g}_{l}^{(2)}(0)$ are plotted as a function of *ρ*.

In conclusion we have shown the single photon emission properties of interface-fluctuation GaN quantum dots as a function of their ambient temperature. The quantum nature of the emission is maintained up to 77 K, although its intensity and purity gradually decreases, due to increased spectral contamination from uncorrelated background emission related to quantum-well states. It is possible that improved temperature characteristics could be achieved by reducing the volume of measured material- perhaps via the fabrication of mesa-like structures, or by local resonant excitation.

### Data Availability

Datasets for the figures in this paper are available from the corresponding author on reasonable request.
